# Simulation-Based Education for Staff Managing Aggression and Externalizing Behaviors in Children With Autism Spectrum Disorder in the Hospital Setting: Pilot and Feasibility Study Protocol for a Cluster Randomized Controlled Trial

**DOI:** 10.2196/18105

**Published:** 2020-06-04

**Authors:** Marijke Jane Mitchell, Fiona Helen Newall, Jennifer Sokol, Katrina Jane Williams

**Affiliations:** 1 Department of Neurodevelopment and Disability Royal Children's Hospital Melbourne Australia; 2 Department of Paediatrics The University of Melbourne Melbourne Australia; 3 Murdoch Children's Research Institute Melbourne Australia; 4 Department of Nursing Research Royal Children's Hospital Melbourne Australia; 5 Department of Nursing Education Royal Children's Hospital Melbourne Australia; 6 Department of Nursing The University of Melbourne Melbourne Australia; 7 Simulation Program Department of Medical Education Royal Children's Hospital Melbourne Australia; 8 Department of Paediatrics, Education and Research Monash University Melbourne Australia

**Keywords:** feasibility studies, autism spectrum disorder, intellectual disability, high-fidelity simulation training, pediatric nursing, child, adolescent, aggression

## Abstract

**Background:**

Children with autism spectrum disorder (ASD) frequently demonstrate aggression and externalizing behaviors in the acute care hospital environment. Pediatric acute care nursing staff are often not trained in managing aggression and, in particular, lack confidence in preventing and managing externalizing behaviors in children with ASD. High-fidelity simulation exercises will be used in this study to provide deliberate practice for acute care pediatric nursing staff in the management of aggressive and externalizing behaviors.

**Objective:**

The purpose of this study is to conduct a pilot and feasibility cluster randomized controlled trial (RCT) to evaluate the effectiveness of simulation-based education for staff in managing aggression and externalizing behaviors of children with ASD in the hospital setting.

**Methods:**

This study has a mixed design, with between-group and within-participant comparisons to explore the acceptability and feasibility of delivering a large-scale cluster RCT. The trial process, including recruitment, completion rates, contamination, and completion of outcome measures, will be assessed and reported as percentages. This study will assess the acceptability of the simulation-based training format for two scenarios involving an adolescent with autism, with or without intellectual disability, who displays aggressive and externalizing behaviors and the resulting change in confidence in managing clinical aggression. Two pediatric wards of similar size and patient complexity will be selected to participate in the study; they will be randomized to receive either simulation-based education plus web-based educational materials or the web-based educational materials only. Change in confidence will be assessed using pre- and posttraining surveys for bedside nursing staff exposed to the training and the control group who will receive the web-based training materials. Knowledge retention 3 months posttraining, as well as continued confidence and exposure to clinical aggression, will be assessed via surveys. Changes in confidence and competence will be compared statistically with the chi-square test using before-and-after data to compare the proportion of those who have high confidence between the two arms at baseline and at follow-up. The simulation-based education will be recorded with trained assessors reviewing participants’ abilities to de-escalate aggressive behaviors using a validated tool. This data will be analyzed using mean values and SDs to understand the variation in performance of individuals who undertake the training. Data from each participating ward will be collected during each shift for the duration of the study to assess the number of aggressive incidents and successful de-escalation for patients with ASD. Total change in Code Grey activations will also be assessed, with both datasets analyzed using descriptive statistics.

**Results:**

This study gained ethical approval from The Royal Children's Hospital Melbourne Human Research Ethics Committee (HREC) on November 1, 2019 (HREC reference number: 56684). Data collection was completed in February 2020. Data analysis is due to commence with results anticipated by August 2020.

**Conclusions:**

We hypothesize that this study is feasible to be conducted as a cluster RCT and that simulation-based training will be acceptable for acute care pediatric nurses. We anticipate that the intervention ward will have increased confidence in managing clinical aggression in children with ASD immediately and up to 3 months posttraining.

**Trial Registration:**

Australian New Zealand Clinical Trials Registry (ANZCTR) ACTRN12620000139976; http://www.ANZCTR.org.au/ACTRN12620000139976.aspx

**International Registered Report Identifier (IRRID):**

DERR1-10.2196/18105

## Introduction

### Background and Rationale

Autism spectrum disorder (ASD), a neurodevelopmental disorder identified in childhood, is characterized by persistent deficits in social communication and social interaction across multiple contexts and restricted, repetitive patterns of behavior or interests [1]. Children diagnosed with ASD, with or without an intellectual disability, have an increased risk of hospitalization [2-5]. They also have longer and more frequent outpatient visits and medications prescribed than children in general [6]. Intellectual disability, one of the most prevalent comorbidities occurring in approximately 40%-70% of individuals with ASD [7-9], has been associated with greater autism symptomology with increased difficulties in communication and social functioning [10].

Exposure to the hospital environment can provide a sensory overload to children with ASD as it is often noisy and brightly lit, with people moving quickly, many unfamiliar people, and long wait times [11]. The surroundings are unfamiliar and usual home routines are not able to be maintained. The hospital environment necessitates that children must communicate with many more people, mostly unfamiliar, than usual. This is particularly challenging and stressful for children with ASD as they innately prefer less social interaction and have more difficulty identifying social cues and understanding the expressed emotions of others. Externalizing behaviors are common in children with ASD when exposed to these stressors and can result in difficult or delayed treatment, increased anxiety for the parent and child, prolonged procedure times, increased health care costs, and poorer health outcomes [2,6,11,12].

The Code Grey procedure is one of a series of emergency response codes used in some Australian hospitals. Code Grey refers to *unarmed aggression* and is called when an individual fails to respond to initial defusing mechanisms undertaken by staff [13]. At The Royal Children’s Hospital (RCH) Melbourne, the numbers of Code Grey activations are rising each year. In 2016, there were 611 recorded Code Grey activations, 1050 in 2018, and 1682 in 2019. In 2016-2017, 36% of Code Grey activations were due to aggression demonstrated by children and young people with ASD, with or without an intellectual disability [14].

### Need for a Trial

Staff training programs designed to teach best practice principles in the management of clinical aggression in pediatric acute care settings is warranted; however, evidence on training effects in general hospital settings is scant [15]. There are a number of reviews of aggression management training programs in psychiatric and mental health settings [16,17]; however, the results cannot be generalized to the acute setting or further extrapolated to the pediatric setting due to the different types of care provided in each facility and prior training of staff [18,19]. In addition, aggression management training programs designed for nursing staff working with children with neurodisabilities are rarely described in the literature. Simulation training may be an effective educational tool to practice de-escalation skills in a high-fidelity situation. Simulation-based education for communication skills has been shown to improve patient safety in a number of studies [20-22]. There is a paucity of literature in the use of simulation-based education to teach de-escalation communication techniques to staff working with children and young people, particularly those with ASD, in the acute care setting.

### Choice of Comparator

A purpose-designed and purpose-built web-based learning package for working with children and young people with ASD and aggression or externalizing behaviors was chosen as the comparator. An online learning package was chosen as it is convenient and flexible in time to complete, yet provides consistent information and allows self-paced learning. The online resource includes an opportunity for learner-determined revision of concepts, promotion of active and independent learning, and ability to link to and explore relevant resources.

### Study Purpose and Trial Design

The purpose of this study is to conduct a pilot and feasibility cluster randomized controlled trial (RCT) to evaluate the effectiveness of simulation-based education for staff in managing aggression and externalizing behaviors of children with ASD in the hospital setting.

This study has a mixed design, with between-group and within-participant comparisons to explore the acceptability and feasibility of delivering a large-scale cluster RCT and to assess trial processes, including recruitment, completion rates, contamination, and outcome measures. Randomization of two hospital wards will be performed with 1:1 sample size per cluster.

### Study Objectives

#### Primary Objective

The study objectives are to conduct a pilot and feasibility study and assess features of the acceptability and feasibility of our pilot design.

##### Population, Intervention, and Comparator

Our study population will include clinical nurses working in an acute care pediatric hospital. The study intervention will be comprised of simulation-based education plus web-based education resources on the management of clinical aggression and externalizing behaviors in children with ASD, with or without an intellectual disability. The study comparator will be web-based educational materials on the management of clinical aggression and externalizing behaviors in children with autism.

##### Outcomes

The following criteria will have to be met to indicate that an RCT is feasible and acceptable as planned:

1. Randomization: more than 10% recruitment rate from ward staff. A total of 160 staff members from the two selected wards will be eligible to participate in the study. We aim to recruit 10 staff members to each arm of the study.
2. Completion: less than 20% attrition rate with survey completion rate of at least 80%; focus group participation rate of at least 50% of total participants.
3. Acceptability: high acceptability of the intervention among participants as indicated by 80% of scores being 4 (good) out of 5 or higher in survey data.
4. Data collection: follow-up survey response rate of at least 30% with acceptability and confidence levels maintained; ward data-collection rate of at least 80% of total shifts during study time in each ward.
5. Low contamination from intervention participants as evidenced by participant report.

#### Secondary Objectives

The secondary objectives and their outcomes are as follows:

1. Confidence and competence: 80% of participants reporting increased confidence levels and positive qualitative comments.
2. Data collection: 80% reporting of the number of Code Grey activations and the number of successful de-escalation episodes not requiring a Code Grey activation with description of context and outcome for each incident; participant use of de-escalation skills during simulations using the English Modified version of the De-escalating Aggressive Behaviour Scale (EMDABS).

### Summary

In summary, the aim of this study is to conduct a pilot and feasibility cluster RCT to evaluate the effectiveness of simulation-based education for acute care hospital staff in managing aggression and externalizing behaviors of children with ASD. We hypothesize that a multisite cluster RCT to evaluate this training format is feasible.

## Methods

### Participants, Interventions, and Outcomes

#### Study Setting

The study setting is a tertiary pediatric hospital, the RCH Melbourne, Australia. Recruitment will be performed from nursing staff from one general medical ward and one general surgical ward, both of similar size and patient complexity. Hospital data indicate that children and young people with autism, with or without intellectual disability, are admitted with similar frequency to these wards. The two included wards also experience similar numbers of aggressive incidents per year requiring a hospital Code Grey response.

#### Eligibility Criteria

Clinical nurses who work in the general medical and surgical wards will be invited to participate in the study. Eligible nurses will be those who are responsible for providing direct clinical care for ward patients. Nurses from these wards are excluded if they are not responsible for direct patient care (eg, care coordinators, advanced practice nurses, and nurses in charge of the shift).

#### Interventions

##### Overview

The training intervention will consist of two components:

1. A web-based learning package, developed using Articulate software (Articulate Global), for the management of aggression and externalizing behaviors in children and young people with autism, with or without intellectual disability, in the hospital setting as prereading.
2. Prereading will be followed by a 1.5-hour simulation-based group education session to manage aggression and externalizing behaviors in an adolescent with autism; this will include two separate simulation exercises, each followed by a facilitated reflective debrief, which explores what the participants did well, what the challenges were, and what they will do differently next time. The first scenario involves an adolescent with autism and aggressive and externalizing behaviors. The second scenario increases in complexity and involves a nonverbal adolescent who has autism and intellectual disability who also demonstrates aggressive and externalizing behaviors. The training will be conducted in the Simulation Centre, conducted by the Simulation Faculty and Code Grey Coordinator, with an actor playing the role of the patient. The parent role in each of the scenarios will be played by a member of the Simulation Faculty. The simulation exercises will be recorded using Sportstec (also known as Studiocode) software.

##### Web-Based Education: Comparator

A web-based learning package is being developed using Articulate software. Content will be written by a study investigator with significant experience working with children with ASD and intellectual disability and their families. The content will be reviewed by autism experts within the Department of Neurodevelopment and Disability, RCH; the Murdoch Children’s Research Institute; and the Department of Paediatrics, University of Melbourne. A consumer representative will also review the content. Modifications will be made from the feedback received. The content will be succinct and will be designed for the learner to complete within 30 minutes. A small number of short multiple-choice questions will be included in the education package to test understanding and application of knowledge.

##### Simulation-Based Education: Training Intervention

A 1.5-hour simulation-based education session, focusing on managing aggression and externalizing behaviors exhibited by a young person with autism in the inpatient setting, is being developed by the study investigators in conjunction with the Simulation Faculty. The RCH Simulation Program curriculum and simulation sessions are designed according to the concepts described by Dieckmann et al [23], utilizing the debriefing framework by Rudolph et al [24]. The simulation scenarios will be reviewed and trialed by members of the Simulation Faculty, with modifications to improve the learning experience made prior to conducting the sessions. The training sessions will be delivered by the Simulation Faculty in the Simulation Centre with assistance from the Code Grey Coordinator. Two separate simulation exercises will be delivered within this 1.5-hour simulation training session.

Participants will be required to complete pre- and posttraining surveys and a follow-up survey 3 months posttraining. Each participant will be asked to create a unique identifier for use in all the surveys, using the first three letters of their first street name followed by the first two numbers of their mother’s birth date.

Nurses randomized in the ward to receive the training intervention will receive the simulation-based training intervention plus web-based training resources. They will be sent an email with instructions on how to access the surveys and the training and the requirements of the study. Participants in this arm will be instructed to complete the web-based training prior to attending the simulation-based education.

Nurses working in the ward randomized to receive the comparator will receive the web-based training resources only. They also will receive an email outlining the requirements of the study with links to the surveys and the training materials.

The intervention arm will be sent an electronic link to reserve their place in one of two simulation training sessions. Each session will have capacity for 5 participants. The comparator arm will be sent a link to access the web-based training. It is expected that the participants will confirm with their Nurse Unit Manager (NUM) whether they are able to be released from ward duties for the duration of the training. Participants will also be instructed via email to complete the pretraining survey prior to commencing the training. The time commitment to complete the web-based education will be 30 minutes, with the simulation education involving a 1.5-hour commitment within working hours.

Staff will complete the simulation training during double staff time; during this time, there are more nurses available to attend training while not compromising patient care. The principal investigator will liaise with the relevant nurse education team members to ensure that nurses have the capacity to complete the simulation-based training and the online training during double staff time. This is the time that education is often scheduled for nurses working on wards. The training was scheduled for late Spring 2019, when it was anticipated that ward activity levels would be lower than during the winter period. The nursing executive and the NUMs of the study wards have agreed to support this study, as have the RCH Simulation Program and the Department of Neurodevelopment and Disability.

##### Training Intervention

A researcher who is not involved with this study will explain the study design to the participants prior to commencement of the prebrief to the simulation training. The researcher will explain that participants will complete a short electronic survey at the completion of the simulation education program. The survey will be completed anonymously with no personal details recorded. The researcher will explain that the simulation sessions will be recorded to enable researchers to view the recordings at a later date to assess the impact of the training on participants’ performances in de-escalating aggressive situations. Only the study investigators will have access to the recordings, which will be deleted once analyzed. The assessment of the recordings will not include any participant identification details. Participants in the simulation-based education intervention will be asked to maintain and hold confidential all information regarding the performance of specific individuals and the details of the specific scenarios. The researcher will check that all participants have completed the pretraining survey. For those who have not, time will be provided for them to complete it in a nearby room with computer access prior to commencing the simulation training.

The Simulation Faculty member facilitating the simulation session will then brief the participants on the objectives of the two simulation exercises. The roles and expectations of both the participants and the instructors will be discussed. The structure of the session and the purpose of the postsimulation debrief will be explained. The participants will be informed that the same professional actor will play the role of the patient in each of the simulation exercises. The second simulation scenario will be more complex than the first, giving the participants the opportunity to extend their skills and build on knowledge learned from the first scenario. Participants will be asked to volunteer for the different roles in each simulation scenario. The Simulation Faculty technologist will orient the participants to the Simulation Centre and simulation equipment prior to commencement of the first simulation scenario.

#### Outcomes

The primary and secondary outcome measures for assessing the acceptability and feasibility of conducting the training intervention are detailed in the Study Objectives section above.

Two simulation scenarios involving an adolescent with autism, intellectual disability, aggression, and externalizing behaviors will be assessed. Study participants will be asked to complete three surveys: a pretraining survey, a posttraining survey, and a follow-up survey.

The purpose of the pretraining survey is to determine participants’ self-perceptions of confidence and competence in managing aggressive and externalizing behaviors in a young person with autism. The pretraining survey incorporates the Confidence in Coping with Patient Aggression Instrument [25], short-answer questions, and free text to assess self-perceived levels of confidence and competence in managing aggression in a young person with autism and intellectual disability as well as acceptability of the simulation- and web-based education. The Confidence in Coping with Patient Aggression Instrument is a one-dimensional, 10-item instrument demonstrating a high degree of internal consistency (Cronbach alpha=.92) and precision (SE 1.5) [25].

The purpose of the posttraining survey is to determine if the training had an impact on participants’ self-perceptions of confidence and competence in managing aggression and externalizing behaviors in a young person with autism. The posttraining survey also incorporates the Confidence in Coping with Patient Aggression Instrument [25], short-answer questions, and free text to assess self-perceived levels of confidence and competence in managing aggression and externalizing behaviors in a young person with autism and intellectual disability as well as acceptability of the simulation- and web-based education. The purpose of the follow-up survey is to determine if the training had a continued impact on participants’ self-perceptions of confidence and competence in managing aggression in young people with ASD.

Each of the simulation training scenarios will be recorded. The recordings will be analyzed by two clinicians, who are experienced in clinical aggression management, using the EMDABS to assess the influence the training had on participants’ performances in de-escalating aggressive situations. A study investigator will train the clinicians in the use of the tool using the training materials provided by Mavandadi et al [26], providing a clear definition of terms and items and the scoring system. Both clinicians will use the tool on a recording of a practice simulation involving only members of the Simulation Faculty and the study investigator and will discuss decision-making processes to ensure consistency and interrater reliability [27].

Knowledge retention by study participants 3 months posttraining, as well as continued confidence and exposure to clinical aggression, will be assessed via surveys similar in content to the pre- and posttraining surveys. Results from the pretraining, posttraining, and follow-up surveys will be linked using the unique identifiers that the participants created prior to completing the pretraining survey.

Data will be collected at the ward level at each shift for the duration of the study to assess the number of aggressive incidents and rates of successful de-escalations for patients with ASD. A short survey will be emailed to Associate Nurse Unit Managers (ANUMs), as well as to any additional nurses who acted in the roles of ANUM or *nurse in charge of shift*, at the completion of the study to explore the enablers and barriers to collecting this data.

Total change in Code Grey activations will also be assessed in total and at the ward level. We will also record rates of data collection.

#### Participant Timeline

**Figure 1:**
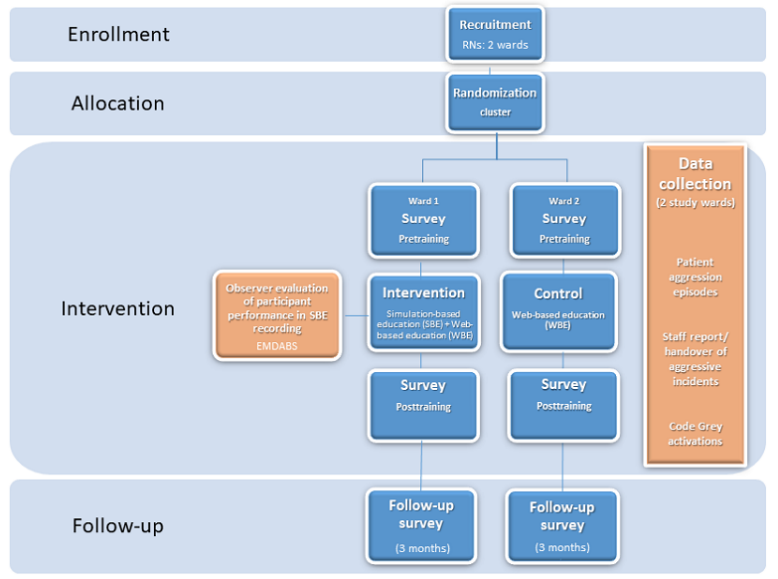
Study design. EMDABS: English Modified version of the De-escalating Aggressive Behaviours Scale; RN: registered nurse.

Figure 1 shows the study design flowchart, including participant recruitment and allocation, intervention components, and follow-up.

#### Sample Size

We plan to recruit 10 staff members to each arm of the study. This sample size, based on our experiences in an earlier study we conducted, is a good size for a pilot and feasibility study to assess recruitment, contamination, and data collection.

#### Recruitment

A study investigator will provide ward-based education about the study, providing clinical nursing staff with information about the study and an opportunity for questions during the double staff time in each ward two times in a 2-3-week period prior to commencement of the study. Hard copies of the study information will also be provided. Following face-to-face information sharing, study information and a request to participate in the study will be forwarded via email to each nurse in the ward by a study investigator. This email will describe the study and outline the participant requirements. It will include a participant information statement and consent form. Two reminder emails will be sent 1 week apart to potential participants to remind them to respond.

The first 10 nurses from each ward who agree to participate in the study and return the consent form will be accepted into the study. Following randomization, participants from each arm of the study will be sent an information email, specific to the group to which they have been randomized, with all the information they require to participate in the study. This email will include links to the relevant resources and surveys as well as links for securing a position into the simulation training and the focus group interviews. Then, 3 months posttraining, all participants will receive an email reminding them to complete the follow-up survey. This email will be resent two times as a reminder to participants. At the completion of the study, participants will receive a letter that provides a summary of the main study findings and thanks them for their participation in the study.

### Assignment of Groups

#### Allocation

A researcher who is not associated with this study and is not based at the study site will use a coin toss to randomize the wards included in the study to either the training intervention or the comparison group. This will occur once recruitment is complete. The first coin toss will identify which ward is being considered (ie, heads for one ward and tails for the other). The second coin toss will decide whether the selected ward is assigned to the intervention arm or to the comparator arm. Once the wards have been allocated, the study investigator will be unblinded and will individually notify participants via email regarding the arm of the study to which they have been allocated.

#### Blinding

Blinding for the study investigators and participants will not be possible. To reduce bias, once data are exported to Microsoft Excel, a researcher who is not involved with the study will code the intervention and comparator groups and remove data columns that relate to the intervention group only, so the person conducting the analysis will be blind to group allocation.

### Data Collection, Management, and Analysis

#### Overview

##### The Kirkpatrick Four-Level Model

The Kirkpatrick Model will be used to measure the impact of the training. This four-level model evaluates training according to (1) reaction, (2) learning, (3) behavior, and (4) results [28-30].

##### 1. Pre- and Posttraining Survey (Kirkpatrick Level 1)

A link to a short, electronic REDCap (Research Electronic Data Capture) survey will be administered to participants’ before and after simulation training, incorporating the Confidence in Coping with Patient Aggression Instrument [25], short-answer questions, and free text. These will be used to assess self-perceived levels of confidence and competence in managing aggression as well as the acceptability of the simulation and web-based training.

##### 2. Follow-Up Survey: 3 Months Posttraining (Kirkpatrick Level 1)

A link to a short, electronic REDCap survey will be emailed to participants 3 months after simulation training incorporating the Confidence in Coping with Patient Aggression Instrument [25], short-answer questions, and free text. These will assess self-perceived levels of confidence and competence in managing aggression as well as the acceptability of the simulation and web-based training.

##### 3. Observer Evaluation of Use of De-escalation Skills Within the Simulation (Kirkpatrick Level 2)

The simulation exercises will be recorded using Sportstec software. Sportstec is video analysis software designed for use in medical simulation research that allows users to interpret and code the multifaceted elements of video while identifying patterns that form a comprehensive picture. The video recordings are securely held on RCH servers and the software license includes use for research purposes. Two clinicians with expertise in the management of clinical aggression will assess the participants’ recorded performances for each scenario using the EMDABS [27,31]. The EMDABS is a one-dimensional, seven-item scale combined with a 5-point Likert scale ranging from 1 (*strongly disagree*) to 5 (*strongly agree*). Performance will be represented by the means of the seven items. The original German-language scale (De-escalating Aggressive Behaviour Scale, DABS) was enhanced and validated by Mavandadi et al [26] to create the EMDABS. This tool demonstrated good interrater reliability (intraclass correlation coefficient=.752) and strong internal consistency (Cronbach alpha=.901). Training materials have been provided by the authors of the EMDABS tool and will be used to train the clinicians in the use of the tool.

##### 4. Ward Patient Aggression Record (Kirkpatrick Level 3)

Episodes of clinical aggression from patients with ASD will be recorded during each shift in participating wards 1 month prior to and 3 months after the training intervention. Numbers of successful de-escalation interactions will be recorded. A short, electronic REDCap survey will be emailed to ANUMs, and to any additional nurses who acted in the role of ANUM, at the completion of the study to explore the enablers and barriers to collecting this data.

##### 5. Code Grey Activations (Kirkpatrick Level 4)

We will conduct a review of numbers and context of Code Grey activations in participating wards for 1 month prior to and 3 months after the training intervention. Strategies to be used by the study team to promote participant retention and completion of follow-up surveys are described in the Recruitment section.

#### Data Management

Participant confidentiality will be strictly held in trust by the study investigators and the sponsoring institutions. All surveys are anonymous so no personal details will be stored. The data from the EMDABS will be entered into a password-protected REDCap account by an administrative assistant. All survey data will be entered directly by participants into the secure REDCap web-based application. Sportstec recordings of the simulation-based education will be deleted once analysis is complete. All data for this study will be stored on a password-protected computer located in the Department of Neurodevelopment and Disability for 5 years. After 5 years, the data will be deleted.

#### Statistical Methods

##### Primary Outcomes

Percentages will be calculated to estimate recruitment, retention, outcomes, survey response rate, focus group participation, and the precision of those estimates. We will treat the Likert-scale scores as categories and dichotomize the 11- and 5-point scale responses. Changes in confidence and competence will be compared statistically with a chi-square test using before-and-after data to compare the proportion of those who have high confidence between the two arms at baseline and at follow-up.

##### Secondary Outcomes

The Code Grey data and daily aggression data will be analyzed using descriptive statistics, including descriptions of the clinical journey for children and young people who trigger more than one Code Grey response. We will report whether aggression de-escalation attempts were recorded during each shift on each ward as planned. The EMDABS data will be analyzed using mean values and SDs to understand the variation in performance of individuals who undertake the training.

### Monitoring

A Data Monitoring Committee is not required, as this is a feasibility and pilot study of short duration and minimal risk. Due to the realism of simulation-based education, participants could potentially become distressed during, or at the completion of, a simulation scenario or the debrief for many reasons. If a member of the Simulation Faculty observes distress in a participant, they will offer them the option to leave the scenario or debrief and will support them both at the time they leave, as well as follow up with them through the following week. In addition, they will be offered the counsel of the RCH Employees’ Assistance Program. In a previous pilot study conducted prior to this work by the authors, distress following simulation-based education was minimal. Immediate support from the Simulation Faculty posttraining was sufficient to reassure a small number of participants who verbalized concern about their performance. No participants required referral to the RCH Employees’ Assistance Program for continued support or follow-up.

It is not expected that the focus groups will cause participants any anxiety or distress. If participants do not feel comfortable answering any of the questions in the focus group, they do not need to answer them. Auditing of data is not required, as this is a feasibility and pilot study of short duration.

### Ethics and Dissemination

#### Research Ethics Approval

The study received ethical approval from the RCH Melbourne Human Research Ethics Committee (HREC) on November 1, 2019 (HREC reference number: 56684).

#### Protocol Amendments

There are no anticipated amendments to the protocol for this pilot and feasibility study.

#### Consent

Participation will be voluntary and consistent with the National Statement on Ethical Conduct of Human Research Section 2.2.9: no coercion or pressure to participate will occur between the study investigators and potential participants [32].

A study investigator will email the participant information statement and consent form to all potential study participants. Nurses in the study wards will be asked within this email to return the consent form to the nominated study investigator if they would like to participate in the study.

All participants will be required to read the participant information statement and sign and return the consent form via email before participating in the study.

Participants in both arms of the study will be required to (1) complete the training intervention, (2) complete the pre- and posttraining surveys, (3) complete the follow-up survey, and (4) participate in a 1-hour focus group interview at the completion of the training.

#### Confidentiality

##### Overview

Participant confidentiality will be strictly held in trust by the study investigators and the sponsoring institutions. Participants in the simulation-based training will be asked to maintain and hold confidential all information regarding the performance of specific individuals and the details of the specific scenarios. Only study investigators will have access to the trial data.

##### Surveys

All surveys are anonymous and will be completed electronically using a web link to the REDCap survey sent to participants via email. While no personal details will be recorded, it is possible that years of clinical experience may potentially identify some participants. Participants will be asked to create their own unique identifier to be used for each survey, so that pretraining, posttraining, and follow-up survey results can be linked. Data stored in the secure REDCap database will be deleted following completion of data analysis.

##### Sportstec Recordings of the Simulation-Based Education

Confidentiality will be maintained by the assessors; no identifying participant information will be kept and the recordings will be deleted once analysis is complete.

##### Ward Patient Aggression Record

Completed daily ward patient aggression records will be stored in a locked filing cabinet in the ward nursing offices and collected daily on weekdays by a study investigator, who will then store the record sheets in a locked filing cabinet in the Department of Neurodevelopment and Disability. No patient names will be recorded on these records, which will only be identified by the patient Medical Record Number (MRN). This information is reidentifiable; however, only the study investigator (MJM) will have access to the data during the study period. The nurse in charge of the shift who completes the record will have access to the record sheets completed that day and will store them in a locked filing cabinet on the ward until they are collected by a study investigator (MJM). This investigator, who is an RCH clinician and works with this population, will access the medical records to locate the aggressive incident. No personal information will be recorded in REDCap; instead, what will be recorded is whether a de-escalation episode was found or not, to check the validity of the reported data as part of the pilot and feasibility study. Data, excluding the patient MRN, will be entered into the secure REDCap database and will be deleted following completion of data analysis. Written records will be shredded and disposed of securely once data has been entered into REDCap.

#### Dissemination Policy

The results of this study will be disseminated to study participants, staff of the study site, and health care professionals through the dissemination plan in Table 1.

**Table 1:** Dissemination plan.

Domain	Activities	Approach	Time frame	Responsibility	Budget
Dissemination objectives	Goal is to present main findings to participants, staff at study site, hospital executive, health care professionals, and consumers	Dissemination options are detailed below for each group	Within 6 months following completion of study	Study investigators	Nil
Identify target audience	Inform study participants	Letter summarizing the main findings	1 month following completion of study	Study investigators	Nil
	Inform staff at study site	Presentation at research forums, department meetings, and ward education session	Within 6 months following completion of study	Study investigators	Nil
	Inform hospital executive	Face-to-face meeting with Executive Director of Nursing and Allied Health and a written summary of findings	Within 2 months following completion of study	Study investigators	Nil
	Inform health care professionals	Publication in relevant journals, presentation at relevant local and international conferences, and webinar about the study and findings for the Department of Neurodevelopment and Disability	Within 1 year following completion of study	Study investigators	Funding will be sought from relevant scholarships and funding sources
	Inform consumers	Blog of main findings presented in plain English on department website	Within 2 months following completion of study	Study investigators	Nil
Key messages	Determine key messages for each target group following completion of study	Adapt content as appropriate	1 month following completion of study	Study investigators	Nil
Sensitivities	Determine sensitivities for each target group following completion of study	Adapt content as appropriate	1 month following completion of study	Study investigators	Nil
Evaluation plan	Evaluate the effect of the different strategies	Analyze *click* and *open* rates for web-based resources and note implementation strategies at the study site following dissemination of results	Within 1 year following completion of study	Study investigators	Nil

## Results

This study gained ethical approval from the RCH Melbourne HREC on November 1, 2019 (HREC reference number: 56684). Data collection was completed in February 2020. Data analysis is due to commence with results anticipated by August 2020.

## Discussion

Aggression demonstrated by children and young people with autism, with or without intellectual disability, is increasing in acute care pediatric hospitals. Nursing staff often feel underequipped to successfully de-escalate or prevent aggression or externalizing behaviors in this population. It is important that acute care nurses working with children with ASD are confident in managing aggression demonstrated by patients in their workplace. This study aims to develop and pilot test a simulation-based training program to upskill pediatric nursing staff who work with children and young people with autism who display aggression and externalizing behaviors.

## Appendix

Multimedia Appendix 1 HREC Pre submission peer review.

## Abbreviations

ANUM: Associate Nurse Unit Manager
ASD: autism spectrum disorder
DABS: De-escalating Aggressive Behaviour Scale
EMDABS: English Modified version of the De-escalating Aggressive Behaviour Scale
HREC: Human Research Ethics Committee
MRN: Medical Record Number
NUM: Nurse Unit Manager
RCH: Royal Children’s Hospital
RCT: randomized controlled trial
REDCap: Research Electronic Data Capture
