# Supplementary material for: Simulation-Based Education for Staff Managing Aggression and Externalizing Behaviors in Children With Autism Spectrum Disorder in the Hospital Setting: Pilot and Feasibility Study Protocol for a Cluster Randomized Controlled Trial
Source: JMIR Res Protoc. 2020 Jun 4;9(6):e18105. doi: 10.2196/18105 (PMC7303837; doi:10.2196/18105)
Supplement: Multimedia Appendix 1 [file resprot_v9i6e18105_app1.docx]

| **Pre-Submission (Peer) Review Proforma** |
| --- |

The purpose of the review is to identify areas for improvement which will ensure the project is scientifically valid. Please also see [Pre-submission (Peer) Reviewer Process](http://www.rch.org.au/uploadedFiles/Main/Content/ethics/Pre-submission%20Peer%20Review%20Process.docx) for more information.

| **PROJECT TITLE** | A pilot and feasibility study of a randomised controlled trial (RCT) to evaluate the effectiveness of simulation-based team training for staff in managing challenging behaviours of children with autism spectrum disorder (ASD) in the hospital setting. |
| --- | --- |
| **Version number & date of Protocol under review** | Version 1  Date: 23/07/2019 |

| **PRINCIPAL INVESTIGATOR** | Marijke Mitchell |
| --- | --- |
| **Position Title** | Clinical Nurse Consultant |
| **Department / Group** | Neurodevelopment & Disability |
| **Institution** | Royal Children’s Hospital |

| **PEER REVIEWER NAME** | Daryl Efron |
| --- | --- |
| **Position Title** | Paediatrician |
| **Department / Group** | Health services Research, Pop Health |
| **Institution** | MCRI |

**PEER REVIEWER DECLARATION:**

- I agree to maintain confidentiality of all matters and documents regarding this project; and
- I am independent of this project; and

I agree that I have no potential conflicts of interest in reviewing this research protocol; **OR**

I declare I have the following potential conflicts of interest:

*Please disclose any actual or potential conflict of interest in the research being reviewed, including any:*

1. *Personal involvement or participation in the research*
2. *Financial or other interest or affiliation, or*
3. *Involvement in competing research*

**Please explain any ‘No’ response and also record any comments regarding required changes or suggestions which could improve the project in the relevant section on the last page of this form.**

| **CRITERIA:** *Using the right column please indicate if each criteria has been addressed, in your opinion* | | **YES**  **NO**  **N/A** |
| --- | --- | --- |
| **Project details:** Has all appropriate information been included?  (Investigator details and project title, protocol version number and date) | | Yes |
| **Research question:** Is there a clearly and precisely defined, answerable question?  Is there a clear aim or objective? | | Yes |
| **Background:** Is the research question an important one? Does the background information provided give a good rationale for why the project is being done? Is the study useful to clinical practice? Is there a real problem/ knowledge gap that needs filling? | | Yes |
| **Plan of Investigation:** | | |
| 1 | **Design:** is the design appropriate to the aim? Will the study address the question being asked and is it likely to produce an answer? | Yes |
| 2 | **Bias and confounding**: Has the study been designed to minimise the risk of bias? Have the investigators adequately accounted for the influence of potential confounders? | Yes |
| 3 | **Randomisation and Blinding:** Where applicable, is enough detail provided on exactly how randomisation and blinding will be achieved, including who is responsible? | See comment |
| 4 | **Sampling issues:** Will the proposed study group be large enough to provide sufficient statistical precision or power, where appropriate? Is there a reasonable justification for the proposed sample size? Will the sample collected be reasonably representative of the population in question? | Yes |
| 5 | **Feasibility:** Is there sufficient evidence to indicate that it will be possible to obtain the numbers required for the study? Is the study feasible in terms of funds, time and other resources? | Yes |
| 6 | **Participants:** Are the criteria for eligibility clear and justified? Have the methods used to identify, approach, recruit and consent participants been clearly and completely described? | Yes |
| 7 | **Intervention or exposure:** Is the intervention or exposure factor clearly described in adequate detail, where appropriate? If the intervention is a drug, are details of dose, delivery, preparation, handling and compliance provided? | Yes |
| 8 | **Procedure plan:** Has an appropriate plan of the study been detailed? Is the estimated duration of the project stated and appropriate? Is it clear how a participant will progress through treatments, procedures, assessments and visits, where applicable? | Yes |
| 9 | **Outcome measures:** Are these appropriate and achievable? Are definitions sufficiently detailed? Is the relevant data being collected on the proposed outcomes? | Yes – see minor comment |
| 10 | **Adverse Events:** Is there an appropriate plan for detecting, managing, recording and reporting defined adverse events, where applicable? | Yes |
| 11 | **Data collection:** are the proposed data collection tools and data management systems appropriate for the project? | Yes – see minor comment |
| 12 | **Analysis:** is there an adequate indication of what analysis will be done on outcome measures to answer the research question? Are the proposed analyses appropriate? | See comment |
| **Project management:** have adequate arrangements been specified for conduct and oversight? | | Yes |
| **Expertise:** Does the research team include (or have access to) all the necessary expertise for the project? | | Yes |
| **Ethical issues:** Have any potential ethical issues been addressed? Are risks to participants minimised? | | Yes |

**Each question, comment, suggestion or requirement should be separately bulleted.**

**Where applicable please reference the section and page number.**

| **General Comments** (Remarks that the investigator does not need to respond to) |
| --- |
| - The project is important, and has high potential to lead on to a properly powered multi-centre RCT which will inform practice improvement. - This protocol was a pleasure to read – really! Background and study rationale well-written, aims are clear, method appropriate and clearly described using PICO framework, and outcome measures for the pilot also appropriate and clearly described. - Great to see use of simulation-based training for management of challenging patient behaviour. - Primary outcomes: 4 Data collection. What is the basis for deeming a 30% follow-up survey completion rate as indicating a RCT is feasible? Seems low. - Sim-based training. Will this be offered during shifts or outside i.e. in staff’s own time. If the former, how will staff relief be arranged and funded? If the latter will they be paid? - Fig 2 – suggest adding a legend - Randomisation sequence generation. Is a statistician really required, given the study will not be blinded? Couldn’t you just flip a coin? |
| **Required Changes** (Points that the investigator must address by either making the required change, or producing a cogent argument against the change) |
| - 9.3 Statistical Methods. Recruitment, retention, survey response etc rates – not sure you can apply 95% CIs meaningfully to such simple statistical counts? - - Primary outcomes. Insufficient detail on analytic strategy wrt between-group comparisons in self-rated confidence and competence. Chi square test is used to compare proportions between groups, so for categorical data. Presume then that plan is to dichotomise the 5-point scale responses? PLs provide detail. - Sec outcomes: “We will report if data is collected on each ward as planned” – unclear. Pls re-word |
| **Suggested Changes** (Points that the reviewer thinks may improve the project. They are not of such importance that they would render the project scientifically invalid/unethical if the investigator did not address the issues) |
| - Patient population of interest – this varies, in both the protocol and surveys, between ASD and ASDS/D. Suggest include any Code Grey in patients with ASD +/- ID. - Appendices 3 and 4: Part B, Q 3: How able are you to intervene physically with an aggressive patient? – I wonder whether respondents may interpret this variably – I’m physically able but would probably do it badly! Not sure what the point of this Q is in relation to constructs of interest as outcomes i.e. confidence and competence – which are both explicitly inquired about in subsequent Qs. - Part C Q 11: How confident do you currently feel in managing aggression? – suggest amend to …..aggression in adolescents with ASD. - Survey completion – suggest use exclusively electronic completion (REDCap). It’s 2019. |
| Good luck with this study |

| **Pre-submission review outcome:** (reviewer to circle) | |
| --- | --- |
| **A** | **No changes required:** take the study forward to submission. |
| ***B*** | **Changes suggested:** at the discretion of the investigator; take the study forward to submission. |
| **C** | **Changes required:** decision about acceptability of subsequent changes at the discretion of the HREC representative. A peer reviewer does not need to review the amended protocol prior to submission. |
| **D** | **Changes and further peer-review required:** decision about acceptability of the subsequent changes at the discretion of the reviewer following a pre-submission peer review of the amended protocol. An additional review & proforma should be completed to document the review of the amended protocol. |

| **Peer Reviewer**  **Signature** | 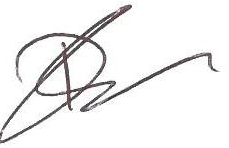 | **Date** | 26/7/19 |
| --- | --- | --- | --- |
